# The intensive care unit balancing act: integrated staffing models for better patient ratios

**DOI:** 10.62675/2965-2774.20260088

**Published:** 2026-03-26

**Authors:** Nicole M. Thomasian, Hayley B. Gershengorn, Hannah Wunsch

**Affiliations:** 1 Department of Anesthesiology, Weill Cornell Medicine New York NY United States Department of Anesthesiology, Weill Cornell Medicine - New York, NY, United States.; 2 University of Miami Miller School of Medicine Miller School of Medicine Division of Pulmonary, Critical Care, and Sleep Medicine Miami Florida United States Division of Pulmonary, Critical Care, and Sleep Medicine, Miller School of Medicine, University of Miami Miller School of Medicine - Miami, Florida, United States.

Modern intensive care units (ICUs) are generally staffed by physicians trained specifically in intensive care (intensivists). However, they can also involve a diverse array of other physicians, advanced practice providers (APPs), and interprofessional team members to facilitate optimal patient care.^(1,2)^ The overall number of patients cared for by an intensivist is primarily dictated by the number of ICU beds in a unit, which can vary dramatically across hospitals and regions.^(3,4)^ However, there can also be large variability day-to-day in demand for beds, and severity of illness of patients.^(5–7)^ Understanding whether patient-to-intensivist ratios (PIRs) affect the quality of care provided and, if so, how to optimize staffing ratios to appropriately care for critically ill patients is essential. Overstaffing may be wasteful, while understaffing may create strain and lead to poorer outcomes. Research on the optimal ratio of PIRs is limited and yields conflicting conclusions.^(3,8–12)^ The safety and effectiveness of different PIRs, however, are likely affected not only by patient case mix but also by the availability of physician trainees, APPs, non-intensivist physicians, and other interprofessional team members, accounting for their education and workload (Figure 1).

**Figure 1:**
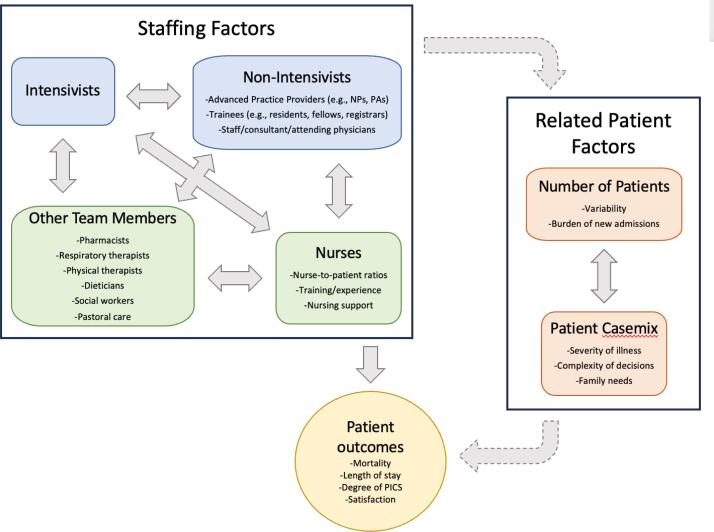
Schematic of the relationship between intensive care unit staffing and outcomes.

NP - nurse practitioner; PA - physician assistant; PICS - post-intensive care syndrome.

Patient-to-intensivist ratios have been measured across various systems and countries. Prior work suggests that the median PIR in the United Kingdom is 8.5 (interquartile range [IQR] 6.9 - 10.8),^(3)^ compared with 10.1 (IQR 7-14) in Australia/New Zealand,^(8)^ and a mean of 9.3 (standard deviation [SD] 3.6) in one sample of United States hospitals^(4)^ and 11.8 (SD 5.7) in another.^(10)^ Multiple aspects of these ratios are worth noting: the median PIRs are relatively similar, and there is wide variability within all these countries. The relationship between PIR and outcomes, specifically hospital mortality, is inconsistent across studies and countries. In the United Kingdom, one study demonstrated a reduced mortality with an average PIR of 7.5 compared with both higher and lower values.^(3)^ However, data from Australia/New Zealand and the US found no such association.^(8–10)^ The U-shaped curve of worse outcomes at both high and low PIRs in the United Kingdom has face validity (Figure 2). The observed increase in mortality at lower ratios is consistent with the volume-outcome relationship, also known as "practice makes perfect", previously demonstrated for mechanically ventilated ICU patients and surgical patients, in which less frequent exposure to a given type of patient or procedure is associated with worse outcomes.^(13–15)^ At higher PIRs, as patient load increases, intensivists may reach a "tipping point" beyond which they are strained. Data on ICU rounds demonstrate the "portfolio problem", or "end of round time compression", in which less time is spent on patients discussed later in a given session.^(16)^ Cognitive, emotional, and physical fatigue mount as patient caseload increases.^(17,18)^ Even the simplest patient comes with added "fixed" work costs such as documentation and brief family updates. Moreover, each additional patient increases the potential for additional "variable" work costs, including the need for bedside procedures, follow-up on diagnostic results, and in-depth family communication and support.

**Figure 2:**
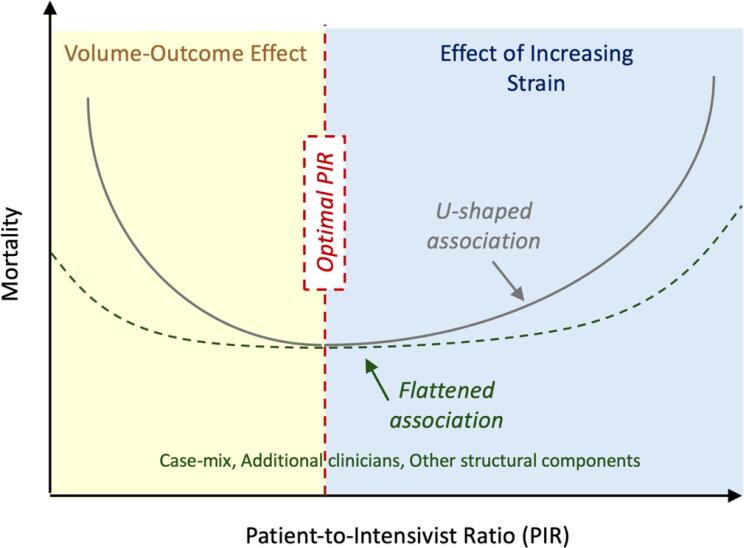
Potential drivers and modifiers of the U-shaped relationship between patient-to-intensivist ratios and patient mortality.* * Data are not real; the figure is meant to be representative of possible associations.

Why might such relationships not be visible in the United States and Australia/New Zealand? One explanation may be that United Kingdom ICUs tend to have a much higher overall severity of illness compared with similar ICUs in the United States or Australia/ New Zealand;^(8,19)^ lower patient illness severity may flatten the curve and shift the "tipping point" for PIR to a higher ratio never reached in practice as the cognitive load and time needed to care for less acutely ill patients may be lower. The idea that a higher PIR may be more tolerable in the United States is supported by a survey of academic intensivists who perceived a cut-off of 14 patients as the point at which the ability to provide effective care (and teach) may begin to degrade.^(20)^ A second possibility is that differences in ICU team structure may help to compensate for a higher intensivist patient load in the US and Australia/New Zealand. Additional bedside physicians and APPs may help to distribute the workload in units with higher PIRs.^(21)^ Research from Australia/New Zealand demonstrates that while the overall PIR (operationalized as the bed-to-intensivist ratio) may vary substantially across units (median 8.0, but IQR 6.0 - 11.4), the patient-to-total physician ratio was both much lower and more consistent (median 3.0, and IQR 2.2 - 4.9).^(22)^ Further, the presence of additional interprofessional team members such as respiratory therapists, clinical pharmacists, dieticians, physical therapists, occupational therapists, social workers, and pastoral care providers may provide key support that reduces intensivist per-patient workload substantially. Data from a study assessing ICUs with interprofessional staff on rounds showed reduced mortality, with or without intensivists.^(2)^

Availability and configuration of ICU teams vary considerably across ICUs; the presence of trainees and APPs is highly variable.^(4,23,24)^ In particular, APPs are relatively common in some countries, such as the US, that deploy them in ICUs to perform tasks and fill roles traditionally considered the purview of physicians, with good outcomes.^(25,26)^ Similarly, the profession of respiratory therapy (and therefore the inclusion of respiratory therapists on an ICU team) is not universal; countries such as Australia/New Zealand do not have respiratory therapists, and a combination of nurses, physiotherapists, and physicians provides this aspect of care.^(27–29)^ Finally, the standard ratio of patient-to-bedside nurse (i.e., 1:1 *versus* 2:1) also varies across ICUs,^(4)^ and having nurses care for fewer patients may allow for a safe shift to higher patient loads for intensivists in some units.^(30–32)^

In an era of constrained resources, staff burnout, and an aging population, research to understand optimal staffing models for the care of critically ill patients remains an urgent concern. Available evidence suggests that PIR may be important, but not in isolation. Instead, PIR must be considered in context: What other physicians and interprofessional team members are available? What is the patient load for other clinicians (i.e., nurses, APPs, respiratory therapists, etc.)? What is the education (e.g., registered *versus* licensed practical nursing degrees) and experience of the ICU clinicians? And, perhaps, how effectively do ICU clinicians function as a team? Finally, we need to document and evaluate staffing ratios in many more countries to better understand the full range of approaches to delivering effective critical care.

## Data Availability

The contents underlying the research text are included in the manuscript.
